# Partial volume correction for improved PET quantification in ^18^F-NaF imaging of atherosclerotic plaques

**DOI:** 10.1007/s12350-017-0778-2

**Published:** 2017-02-07

**Authors:** Jacobo Cal-Gonzalez, Xiang Li, Daniel Heber, Ivo Rausch, Stephen C. Moore, Klaus Schäfers, Marcus Hacker, Thomas Beyer

**Affiliations:** 1 Center for Medical Physics and Biomedical Engineering, Medical University of Vienna, General Hospital Vienna, Waehringer Guertel 18-20/4L, 1090 Vienna, Austria; 2 Division of Nuclear Medicine, Department of Biomedical Imaging and Image-guided Therapy, Medical University of Vienna, Vienna, Austria; 3 Division of Nuclear Medicine, Department of Radiology, Harvard Medical School and Brigham and Women’s Hospital, Boston, MA USA; 4 European Institute for Molecular Imaging, University of Münster, Münster, Germany

**Keywords:** ^18^F-fluoride, partial volume correction, PET/CT imaging of atherosclerotic plaque

## Abstract

**Background:**

Accurate quantification of plaque imaging using ^18^F-NaF PET requires partial volume correction (PVC).

**Methods:**

PVC of PET data was implemented by the use of a local projection (LP) method. LP-based PVC was evaluated with an image quality (NEMA) and with a thorax phantom with “plaque-type” lesions of 18-36 mL. The validated PVC method was then applied to a cohort of 17 patients, each with at least one plaque in the carotid or ascending aortic arteries. In total, 51 calcified (HU > 110) and 16 non-calcified plaque lesions (HU < 110) were analyzed. The lesion-to-background ratio (LBR) and the relative change of LBR (ΔLBR) were measured on PET.

**Results:**

Following PVC, LBR of the spheres (NEMA phantom) was within 10% of the original values. LBR of the thoracic lesions increased by 155% to 440% when the LP-PVC method was applied to the PET images. In patients, PVC increased the LBR in both calcified [mean = 78% (−8% to 227%)] and non-calcified plaques [mean = 41%, (−9%-104%)].

**Conclusions:**

PVC helps to improve LBR of plaque-type lesions in both phantom studies and clinical patients. Better results were obtained when the PVC method was applied to images reconstructed with point spread function modeling.

**Electronic supplementary material:**

The online version of this article (doi:10.1007/s12350-017-0778-2) contains supplementary material, which is available to authorized users.

## Introduction

Cardiovascular diseases (CVDs) are frequently caused by the rupture of a vulnerable atherosclerotic plaque, thus, resulting in thrombotic occlusion or distal embolization.^1^,^2^ Early calcium deposits are common constituents of atherosclerotic plaques, and, therefore, serve as a surrogate marker for atherosclerosis.^3^ CT imaging has been used to assess the severity of vascular calcification and the atherosclerotic plaque burden.^4^ However, CT calcium scores do not represent the pathophysiologic behavior of plaques and, therefore, do not correlate well with cardiovascular risk factors.^5^

In view of the similarities between calcification extent and osteogenesis,^3^ ^18^F-NaF uptake in PET has been described as a marker of ongoing calcium deposition in vulnerable plaque in the carotid arteries.^6^–^10^ These studies, however, showed significant discrepancies between PET and CT findings. While the areas with positive PET uptake correlated with the CT-based calcification, only a fraction of the calcifications identified on CT images presented a significant ^18^F-NaF uptake. Recently, Fiz et al. demonstrated an inverse correlation between calcification and ^18^F-NaF uptake of plaques in the carotid arteries in a cohort of 64 patients.^11^ These studies share one potential limitation. Due to the small size of the vulnerable plaques, which is comparable to the typical spatial resolution of PET, partial volume effects (PVE) may arise and compromise the quantification accuracy of ^18^F-NaF PET uptake in plaques.

Numerous approaches have been proposed for partial volume correction (PVC) in nuclear medicine applications.^12^–^16^ These techniques can be divided into two main categories: within-reconstruction and post-reconstruction methods,^12^ which include projection-based methods, such as the local projection (LP) method proposed for SPECT^17^,^18^ and for pre-clinical PET.^19^

While numerous studies have demonstrated the importance of PVC in neurology, psychiatry, oncology, or cardiology,^16^ few studies have addressed this question regarding PET quantification of vulnerable atherosclerotic plaques, while merely focusing on [^18^F]fluorodeoxyglucose (FDG).^2^,^20^ More recently, Huet et al. stated the importance of implementing advanced PVC methods for a proper assessment of plaque inflammation or calcification with PET.^21^

The aim of this work was to evaluate a PVC method for clinical PET imaging of atherosclerotic plaques. More specifically, this was done by validating the LP method^17^,^18^ on phantom scans and subsequently applying it to clinical ^18^F-NaF PET images of patients with plaques in the carotid or aortic arteries.

## Materials and Methods

### Partial Volume Correction Method

**Figure 1:**
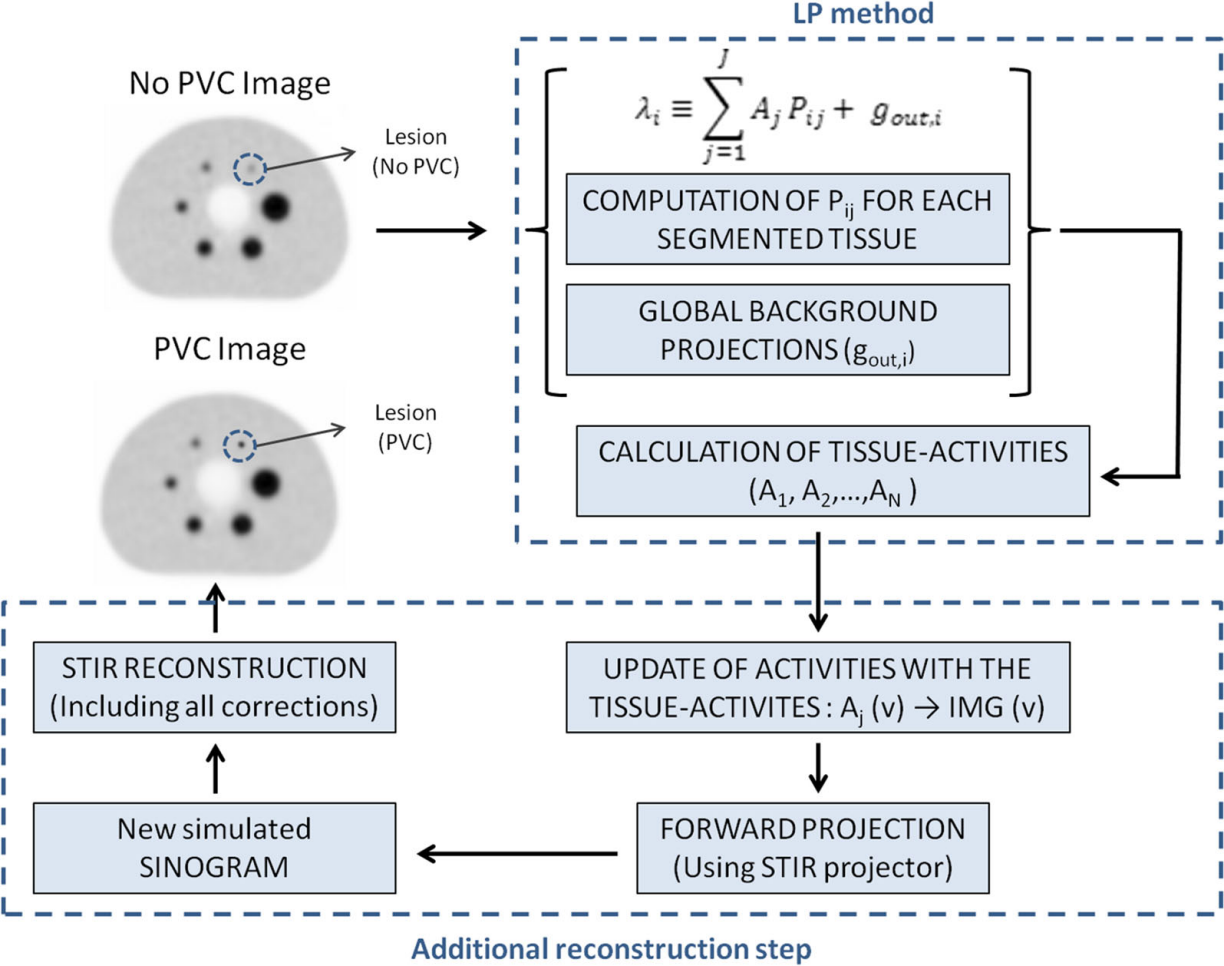
Illustration of the procedure employed to improve the quantification of the reconstructed image within the VOI using the activities computed with the LP method. First, the LP method is applied to obtain the PVC tissue activities of each segmented tissue *j* and voxel *v* within the VOI. Then, the original activities in each voxel within the VOI, IMG (v), are substituted with the LP-based tissue activities, *A*_*j*_ (v). The resulting image is forward-projected to obtain a simulated sinogram, which is then reconstructed with STIR, yielding a simulated PVC image

Consider J different tissue compartments, including the lesion of interest, within a small VOI delineated in the object as shown in Figure 1a (for *J* = 2). The measured emission projection counts for each sinogram bin, λ_*i*_, can be modeled as the sum of the projection counts from each of the *j*-segmented tissue (with unit activity concentration), scaled by their respective activity, plus the counts originating from the global background outside the VOI, as shown in Eq. (1):

1 $$ \uplambda_{i} \equiv \mathop \sum \limits_{j = 1}^{J} A_{j} P_{ij} + g_{{{\text{out,}}i}}, $$

where λ_*i*_ are the expected counts per sinogram bin, *i*, *A*_*j*_ the activity for each segmented tissue j within the VOI, *P*_*ij*_ is the resolution-blurred tissue shape function for tissue *j* and sinogram bin *i*, and $g_{{{\text{out,}}i}}$ represents the background counts coming from the region outside the VOI in each sinogram bin *i*. Note that the indices *i* and *j* increase through the sinogram bins in the projected space and the segmented tissues in the image space, respectively. The tissue activities *A*_*j*_ can be determined by fitting the measured projection data to the model in Eq. (1). Further details can be found in Appendix 1. These tissue activities (*A*_*j*_) can be used as a prior in a new reconstruction procedure, which is in this study performed using the software for tomographic image reconstruction (STIR).^22^ This step leads to a locally “partial volume corrected (PVC)” PET image, with improved visual appearance and quantification of the lesions of interest. A schematic view of the procedure is presented in Figure 1b.

- First, the system matrix values (*P*_*ij*_) for each tissue and the global background *g*_out,*i*_ that affects the VOI activity estimates are computed.
- Later, the LP activities for each segmented tissue are obtained, and the activity in each voxel within the VOI is substituted with the tissue activities obtained with the LP method.
- The resulting image is forward-projected using the STIR projector. A realistic physical modeling of the system is included within the projector, with attenuation and scatter estimates from the anatomical CT image and realistic Poisson noise (taking into account the activity concentration and the acquisition time) included into the forward projection. The scatter was estimated using a version of the Single Scatter Simulation (SSS) algorithm available in STIR.^22^ This step results in realistic “simulated” projection data for the given acquisition.
- The resulting sinogram is reconstructed using a conventional “Ordered Subsets Expectation Maximization” (OSEM) algorithm,^23^ available within the STIR library, with 5 iterations and 21 subsets. No further changes of the LP activity values were made in the new reconstruction procedure. Scatter and attenuation corrections were included into the iterative reconstruction algorithm as additive and multiplicative terms to the estimated data, respectively. This additional reconstruction procedure yields the PVC image.

The above-described procedure for PVC was employed in all the phantom and patient studies. In all cases, the standard PET images were obtained using the vendor software. Two different reconstruction algorithms were used for the reconstruction of the standard PET images: ordered subsets expectation maximization (OSEM) algorithm^23^ and an OSEM reconstruction with point spread function modeling (PSF).^24^ All the relevant physical corrections (attenuation, scatter, normalization, decay, dead time) were included in the vendor OSEM and PSF algorithms. After reconstruction, the LP method for PVC was applied, and the LP activity values were used in an additional STIR reconstruction in order to obtain the PVC image.

### Phantom Evaluations

**Figure 2:**
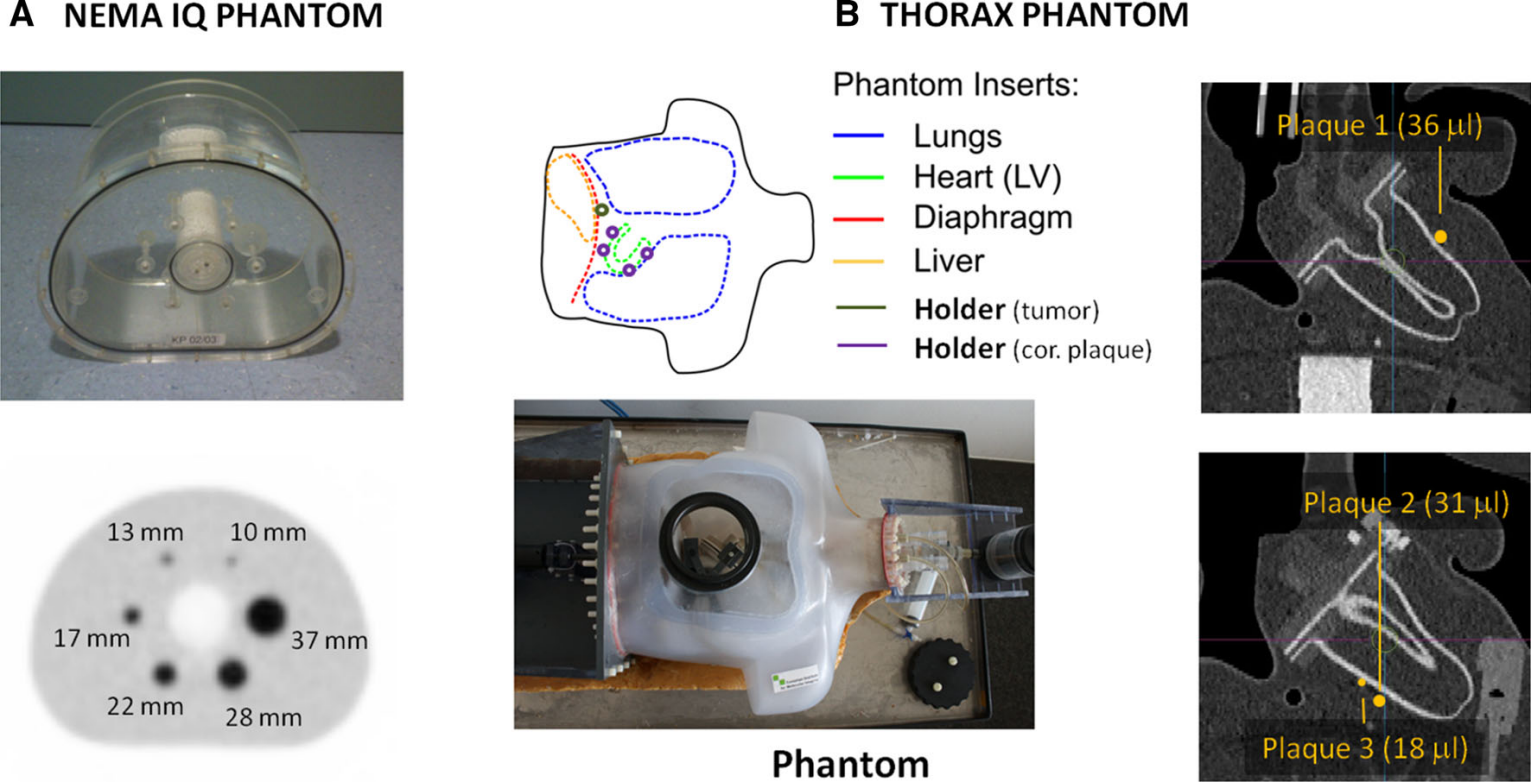
**a** Photograph (*top*) of the NEMA NU2-2012 IQ phantom used for the evaluation and transaxial PET image plane (*bottom*) with the six hot spheres and inner sphere diameter indicated. **b** schematic design of the thorax phantom compartments (*top*), photograph (*bottom*), and positions where the three plaque-type lesions were located (*right*)

The clinical implementation of the LP method was evaluated using acquisitions of a NEMA NU2-2012 IQ phantom^25^ and a human-sized thorax phantom.^26^ The NEMA phantom contains six fillable spheres with internal diameters of 10, 13, 17, 22, 28, and 37 mm (Figure 2a) with an experimental lesion-to-background ratio (LBR) of 4.95, which is in accordance with the LBR values recommended by the NEMA NU-2 2012 protocol for the measurement of image quality (two acquisitions with LBR 4:1 and 8:1, respectively^33^). The thorax phantom has three spherical “plaque-type” lesions of 36, 31, and 18 mm^3^ inserted (Figure 2b) with a LBR of 70:1, following the values suggested in Delso et al.^27^

NEMA IQ phantom data and the thorax phantom were acquired using a Biograph true-point true-view (TPTV) PET/CT^28^ and Biograph mCT PET/CT system, respectively.^29^ The parameters for the acquisition and the reconstruction of the data are summarized in Table 1. OSEM and PSF algorithms, both available from the vendor software, were used for the reconstruction of the acquired images.

**Table 1:** Acquisition and reconstruction parameters for the phantom acquisitions performed in this work

	ACQ time (minute)	Backg. act (kBq/mL)	LBR	Recons methods	Matrix size	Postfiltering (5-mm Gaussian)
IQ phantom	20	4.8	4.95:1	OSEM	336 × 336 × 109	✔
						×
				PSF		
Thorax phantom	10	4.0	70:1	OSEM	400 × 400 × 109	✔
						×
				PSF		

The voxel size used in the reconstructions was 2.03 × 2.03 × 2.03 mm^3^ in all cases

### ^18^F-NaF PET/CT Patient Studies

The LP-based PVC method (Figure 1) was applied retrospectively to a cohort of patients with multiple myeloma, who underwent ^18^F-NaF PET/CT whole-body imaging to characterize bone lesions. In this work, we evaluated the ^18^F-NaF PET uptake in the carotid or ascending aortic arteries. In total, 17 patients (12 male, 5 female, mean age: (64 ± 9) years, range: (47-77) years) with at least one positive plaque were analyzed. Plaques were classified as calcified (HU > 110 within the plaque) and non-calcified (HU < 110), and calcification was defined as the area with a minimum density of 110 HU on CT. Given the fact that a low-dose attenuation-corrected CT (AC_CT) image was used for anatomical reference, a comparatively low HU value,^10^,^11^,^30^ was used as a threshold level for the definition of calcified plaque. In total, 51 calcified (HU > 110 within the plaque) and 16 non-calcified plaque lesions (HU < 110) were analyzed. This retrospective study was approved by the Institutional Ethics Committee and was in accordance with the 1964 Helsinki declaration and its later amendments or comparable ethical standards.

All scans were performed on a Biograph TPTV system.^27^ The patients were injected with (4.3 ± 1.0) MBq/kg (range: 3.1-6.0 MBq/kg) of ^18^F-NaF. The post-injection delay interval was (56 ± 12) minute (range: 34-70 minute), and the PET acquisition time was 2 minute per bed position. 3D PET data were reconstructed using a PSF reconstruction with resolution modeling available from the vendor (4 iterations, 21 subsets). One-bed position image centered in the head-neck region, with 336 × 336 × 109 matrix size and 2.03 × 2.03 × 2.03 mm^3^ voxel size, was obtained for each patient.

### Segmentation Methods

In the cases of calcified plaques and NEMA spheres, the segmentation of the local VOI was performed using a low-dose CT image (512 × 512 × 109 voxels and 1.37 × 1.37 × 2.03 mm^3^ per voxel) co-registered to the PET image. A threshold-based segmentation of the PET image was performed in non-calcified plaque lesions and the thorax phantom data. This segmentation was made using the 3D isocontour half-way between the maximum voxel activity and the mean background activity, as defined by Boellaard et al.^31^ The dependence of the PVC results on the method used for segmentation was evaluated with NEMA IQ phantom acquisitions.

### Data Analysis

*Phantom data* The quantification accuracy was evaluated by measuring the LBR and the relative change of LBR (ΔLBR) after applying the PVC method, for each hot sphere and for each plaque-type lesion. The LBR for each hot lesion was measured using the maximum voxel activity within the sphere (LBR_max_) and the mean activity within a 3D isocontour at 50% of the maximum voxel activity adapted to the mean background activity (LBR_A50_). The ΔLBR was calculated as

2 $$ \Delta {\text{LBR}} (\% ) = \frac{{{\text{LBR}} \left( {\text{PVC}} \right) - {\text{LBR(noPVC}})}}{\text{LBR(noPVC)}} \cdot 100, $$

where LBR(PVC) is the lesion-to-background ratio after applying the PVC (measured from the tissue activities obtained with the LP method or from the PVC image) and LBR(noPVC) is the lesion-to-background ratio measured in the image reconstructed with the vendor software (OSEM or PSF). The activity of the background region, on other hand, was determined by drawing several VOIs in uniform regions.

*Patient data* The maximum HU value within the plaque was evaluated from the CT images. All plaque lesions were classified into four groups: non-calcified (HU < 110), light calcified plaque (110 < HU < 210), medium calcified plaque (210 ≤ HU < 550), and heavy calcified plaque (HU ≥ 550). For calcified plaques, the volume was determined from the CT image by defining all the voxels within the plaque. For non-calcified plaques, we measured the volume of the segmented plaque lesion from the PET threshold-based segmentation. From the PET images, the chosen figures of merit were the LBR using the maximum (LBR_max_) and mean (LBR_mean_) pixel values within the segmented plaque lesion, and the ΔLBR after applying the partial volume correction [Eq. (2)]. The background region was depicted in an arterial region where neither calcium deposition nor increased ^18^F-NaF uptake was detected. This region was placed between 10 mm and 15 mm below the location of the plaque lesion. The LBR and ΔLBR for each group of patients were reported as mean ± SD.

The dependence of the LBR_max_ and ΔLBR_max_ on the density of the plaque (in HU) and the segmented volume was evaluated by fitting the data to the following analytical expression:

3 $$ y = \frac{A}{x} + B, $$

where the dependent variable “*y*” is LBR or ΔLBR and the independent variable “*x*” is the density or the segmented volume of the plaque. *A* and *B* are the fitting parameters. The first term of the equation represents the non-linear dependence close to *x* = 0 (small volume or low HU value of the plaque), while the second term represents the uniform LBR or ΔLBR values observed for large, heavy plaques. Pearson and Spearman coefficients were evaluated to test correlations between the measured variables, and a one-sided paired *t* test was used to evaluate statistical significant changes in the LBR_max_ and ΔLBR_max_ values obtained with the PSF, PSF + PVC, and LP methods.

In 6 of 17 patients, a small misalignment was observed between the PET and the CT image volumes in the carotid region. In these cases, we performed an additional manual fine-tuning of spatial alignment of the images following a rigid affine translation.

## Results

### Validation of the PVC Method and the Segmentation Approaches

**Figure 3:**
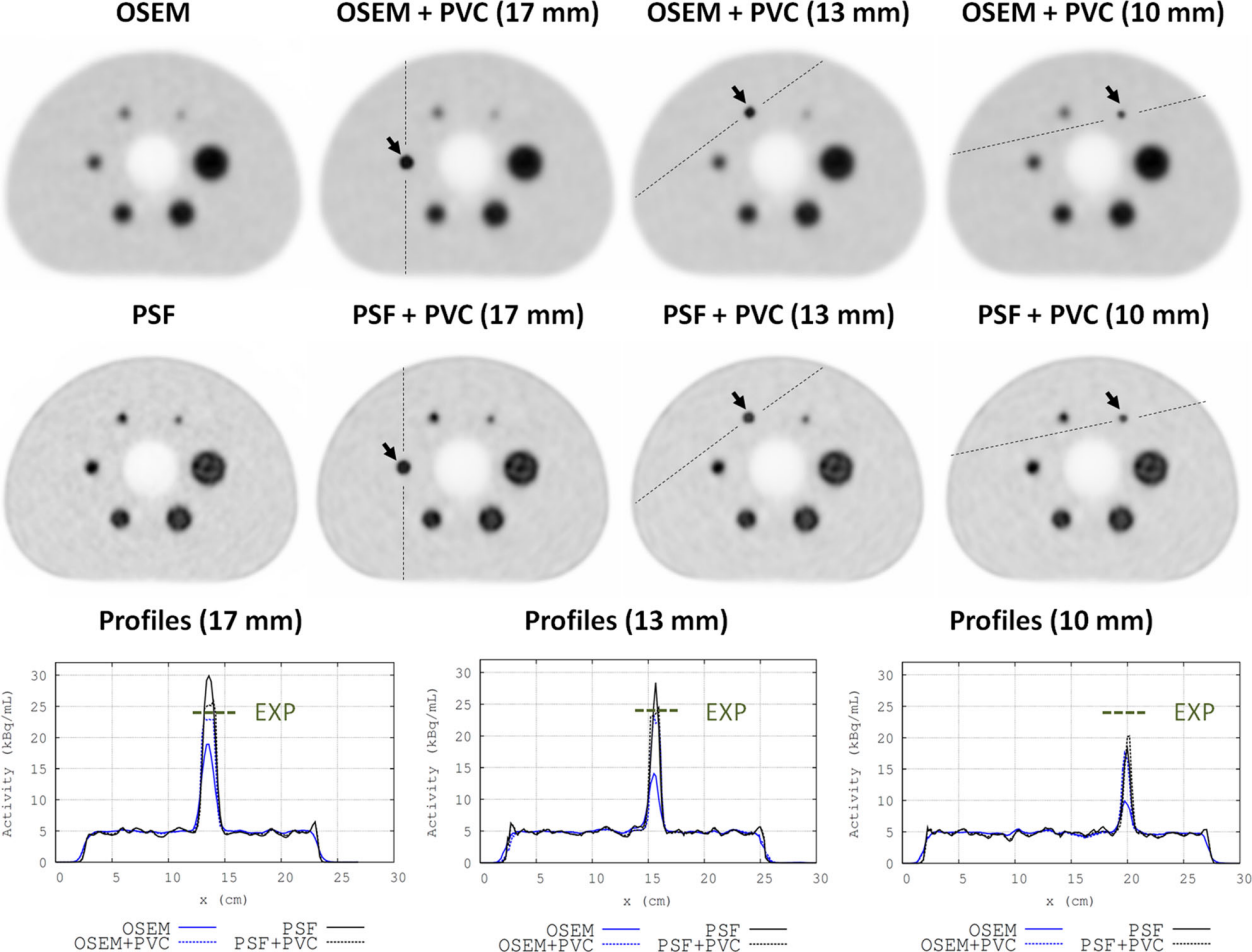
OSEM (*top*) and PSF (*center*) reconstructions of the NEMA IQ phantom. From left to right: standard images reconstructed with the vendor software, PVC image reconstructed with STIR software using the LP tissue activities corresponding to the 17-mm-diameter sphere, the 13-mm sphere, and the smallest 10-mm sphere. Bottom: Activity profiles through the 17-, 13-, and 10-mm-diameter spheres, as depicted in the PVC images. These profiles were obtained from images reconstructed with an OSEM algorithm (*blue*), with (*dashed line*) and without (*solid line*) local PVC, and from PSF images (*black*), with (*dashed*) and without (*solid*) PVC. The measured activity for the spheres was 23.8 kBq/mL, and is marked as a EXP in the plots. The PVC images (*dashed lines*) showed activity values for the spheres close to the measured ones

Figure 3 shows the comparison of the standard OSEM and PSF reconstructions with their corresponding PVC images, for the acquisitions of the NEMA IQ phantom. The local PVC performance is illustrated for the 10-, 13-, and 17-mm spheres. The activity profiles across these spheres are shown in Figure 3(bottom). The quantification of the spheres was significantly improved when the PVC method was applied.

Table 2 summarizes the performance of the PVC method by means of the dependence of the LBR_max_ and LBR_A50_ values on the sphere size (for the NEMA IQ phantom). The LBR values were obtained from the standard images (OSEM and PSF columns) and from the PVC images obtained using the CT-based (OSEM + PVC − CT, PSF + PVC − CT) and the PET-based (OSEM + PVC − PET, PSF + PVC − PET) segmentation approaches. The three columns to the right provide the sphere-to-background ratio obtained using the segmented tissue activities obtained with the LP method (LP − CT and LP − PET, see Figure 1B step 2) and the experimental values measured in a well counter (EXP). The quantification accuracy for the spheres was significantly improved when PVC was applied, yielding LBR_max_ and LBR_A50_ values close to the reference value (EXP).

**Table 2:** LBR_max_ and LBR_A50_ values measured for each sphere of the NEMA IQ phantom in images reconstructed without (OSEM, PSF) and with PVC, obtained using the CT-based (OSEM + PVC − CT, PSF + PVC − CT) and the PET-based (OSEM + PVC − PET, PSF + PVC − PET) segmentation approaches

Sphere diameter (mm)	OSEM reconstructions								
	LBR_max_			LBR_A50_			LP − CT	LP − PET	EXP
	OSEM	OSEM + PVC − CT	OSEM + PVC − PET	OSEM	OSEM + PVC − CT	OSEM + PVC − PET			
37	5.19	5.07	5.15	4.29	4.34	4.61	4.83	4.88	4.95
28	4.94	5.11	5.40	3.99	4.19	4.65	4.74	4.91	
22	4.83	5.25	5.37	3.85	4.26	4.56	4.95	4.99	
17	4.51	5.55	5.76	3.46	4.56	4.73	4.84	5.22	
13	3.43	4.66	4.93	2.66	3.92	4.03	4.68	4.58	
10	2.13	3.46	4.05	1.88	2.87	3.18	3.63	3.78	

Sphere diameter (mm)	PSF reconstructions								
	LBR_max_			LBR_A50_			LP − CT	LP − PET	EXP
	PSF	PSF + PVC − CT	PSF + PVC − PET	PSF	PSF + PVC − CT	PSF + PVC − PET			
37	6.40	5.53	5.54	5.21	4.96	4.88	5.26	5.17	4.95
28	6.31	5.56	5.64	4.68	4.91	4.89	5.29	5.26	
22	6.38	5.66	5.76	4.29	4.48	4.94	5.09	5.39	
17	6.59	5.51	5.79	4.79	5.24	4.92	5.18	5.45	
13	6.26	5.49	5.87	4.21	4.79	4.68	5.08	5.37	
10	4.41	4.91	5.28	3.11	3.73	3.98	4.73	4.76	

On the right of the table, we also show the LBR values obtained using the tissue activities computed by the LP method (LP − CT and LP − PET)

EXP are the experimental values measured in the well counter. Note that the reported LP values resulted directly from the tissue activity estimates obtained from step 2 of Figure 1b, with no further image reconstruction

**Figure 4:**
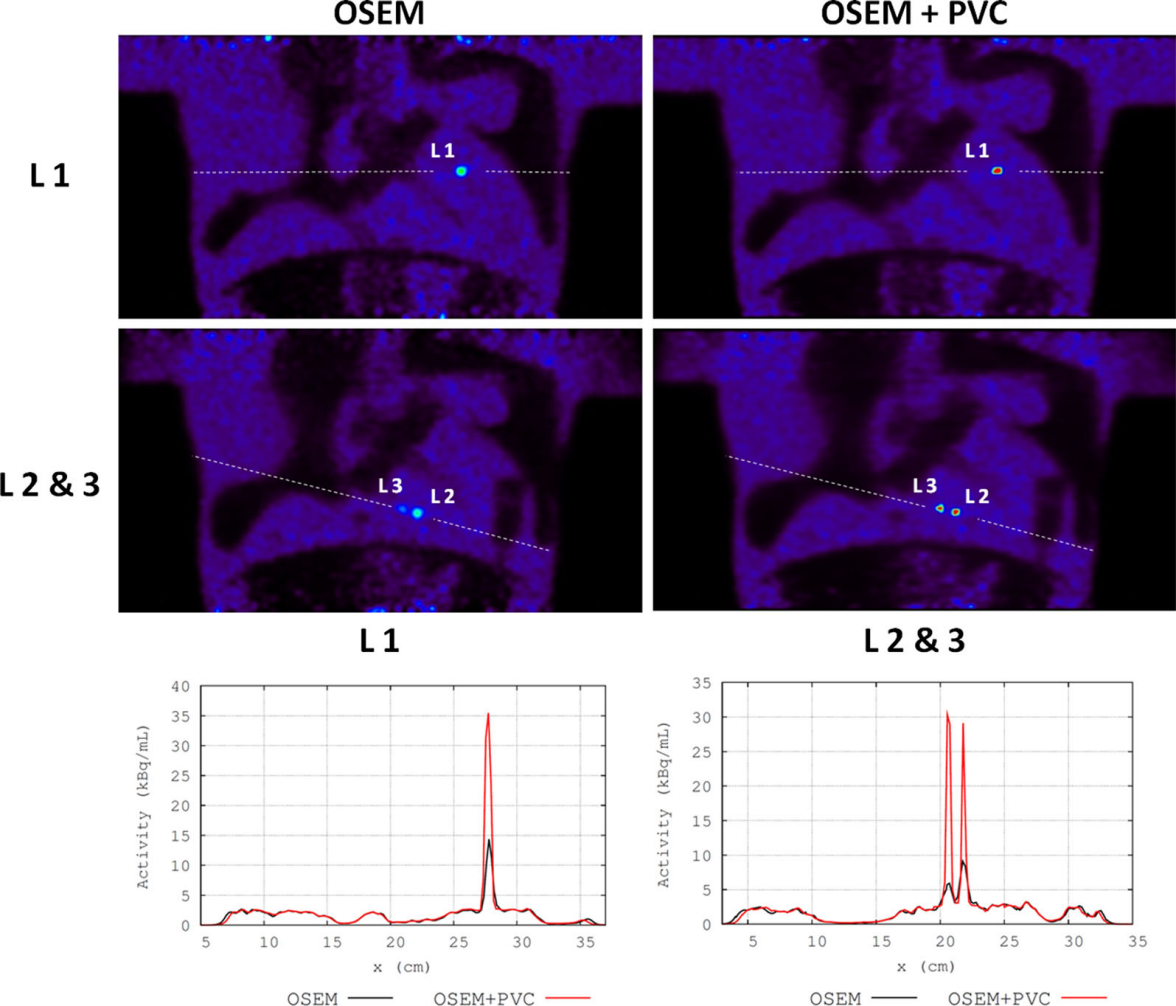
*Top* Coronal views of OSEM (*left*) and OSEM + PVC (*right*) reconstructions of the thorax phantom centered in the plaque-type lesion with 36 mm^3^ volume (L1). *Center* Coronal views centered in the two other plaque lesions, with 31 and 18 mm^3^ volume (L2 and L3, respectively). *Bottom* Activity profiles through L1 (*left*), and L2 and L3 (*right*), as depicted in the images, for the standard OSEM images obtained with the vendor software (*black*) and for the OSEM + PVC images (*blue*)

The performance of the PVC method for the plaque-type lesions in the thorax phantom is summarized in Table 3. Figure 4 shows coronal views of OSEM and OSEM + PVC reconstructions of the thorax phantom with the three plaque-type lesions. Similar results were obtained for the PSF reconstructions. A significant increase in the LBR_max_ and LBR_A50_ values was observed when PVC was applied to the images (ΔLBR_max_ values between 115% and 328% for the three plaque lesions). Furthermore, even larger ΔLBR_max_ values than the ones reported above were observed when comparing the LBR values in the uncorrected images with the tissue activities obtained from the LP method (values between 155% and 475%).

**Table 3:** LBR_max_ and LBR_A50_ values measured for each plaque-type lesion in the thorax phantom in images reconstructed without (OSEM, PSF) and with PVC (OSEM + PVC, PSF + PVC)

Volume lesion (mm^3^)	OSEM Reconstructions					
	LBR_max_ (image)		LBR_A50_ (image)		LP	EXP
	OSEM	OSEM + PVC	OSEM	OSEM + PVC		
36	7.31	28.8	6.00	12.6	25.7	70.0
31	6.02	19.2	5.35	8.59	27.7	
18	3.37	8.86	2.99	4.27	9.45	

	PSF reconstructions					EXP
	LBR_max_ (image)		LBR_A50_ (image)		LP	
	PSF	PSF + PVC	PSF	PSF + PVC		
36	11.4	24.5	9.28	14.4	29.1	70.0
31	8.49	20.0	7.71	9.85	27.7	
18	3.82	16.3	3.58	9.53	20.6	

On the right of the table, we also show the LBR values obtained using the tissue activities computed by the LP method (LP) and the experimental values measured in the well counter (EXP)

### Evaluation of Atherosclerotic Plaque

**Figure 5:**
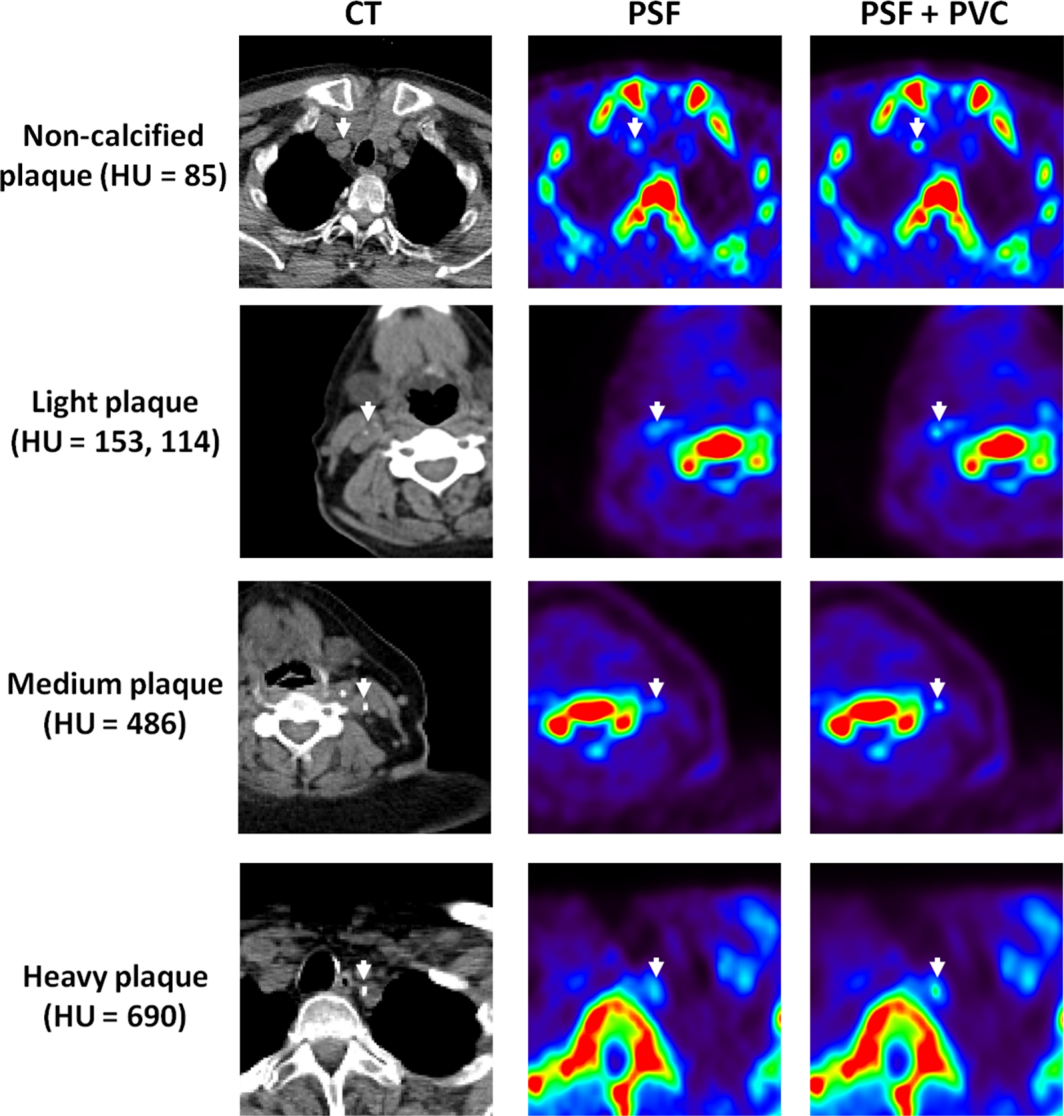
From *left* to *right*: CT, standard OSEM and PVC reconstructions of patients with atherosclerotic plaque in the carotids (*arrows* in CT images). From *top* to *bottom* we show images of patients with non-calcified plaque (HU < 110), light plaque (110 < HU < 210), medium (210 < HU < 550), and heavy plaque (HU > 550) accumulation

**Figure 6:**
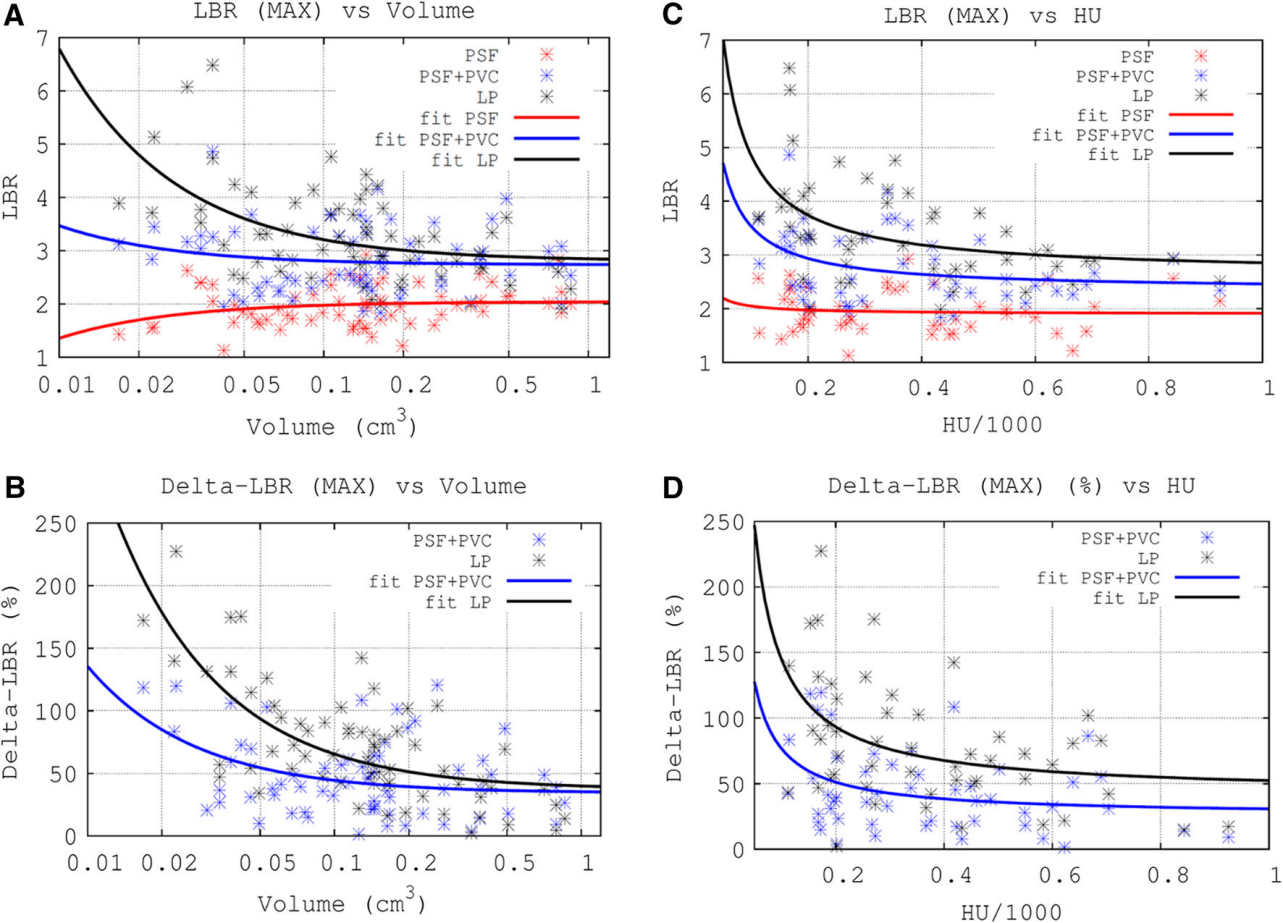
**a** Lesion-to-background ratio (LBR) obtained using the maximum pixel value within the plaque versus the volume of the plaque. **b** Activity recovered after applying the PVC method versus volume of the plaque. **c** Lesion-to-background ratio (LBR) obtained using the maximum pixel value within the plaque versus the HU value of the plaque (only calcified plaques). **d** ΔLBR after applying PVC versus the HU of the plaque (only calcified plaques). The empirical fits of the data to the function $y = A/x + B$, for each of the evaluated methods, are also shown. Note, the segmented volume in panels A and B is represented using a logarithmic scale

Figure 5 shows transaxial images of patients with calcified and non-calcified plaque in the carotids. After PVC, both plaque uptake and delineation of the calcified plaques improve. More specifically, a significant increase of the LBR_max_ was observed in both calcified [mean = 78%, (−8% to 227%)] and non-calcified plaques [mean = 41%, (−9% to 104%)], when the LP method was applied. The relation between LBR_max_ and ΔLBR_max_ with the plaque segmented volume is presented in Figure 6a, b. As expected, the ΔLBR_max_ increases when the volume of the plaque decreases. Figure 6c, d shows the dependence of the LBR_max_ and ΔLBR_max_ on the density of the plaque for calcified plaque lesions. In that case, the LBR_max_ does not demonstrate a significant dependence on plaque density in the absence of PVC. However, we observe a higher LBR for lighter plaques for the case when PVC is applied. This observation is reinforced when the identified plaques are classified into the four groups mentioned above (see materials and methods, data analysis), as can be seen in Tables 4 and 5. Note that the empirical fits in Figure 6 are presented solely to guide the eye of the readers; they do not imply a theoretical dependence, following the fitted function, of the LBR and ΔLBR values with the volume or the HU of the plaque.

**Table 4:** Lesion-to-background ratio (LBR_max_ and LBR_mean_) obtained from the standard PET image reconstructed with the vendor software and PSF algorithm (PSF), the PVC image (PSF + PVC), and from the tissue activities obtained with the LP method (LP)

Plaque type	# lesions	Volume (mm^3^)	LBR_max_ (image)		LBR_mean_ (image)		LP
			PSF	PSF + PVC	PSF	PSF + PVC	
Non-calcified plaque (HU < 110)	16	499 ± 323	2.0 ± 0.3	2.94 ± 0.6	1.7 ± 0.2	2.4 ± 0.4	2.7 ± 0.5
Light plaque (110 < HU < 210)	15	53 ± 34	2.0 ± 0.4	3.1 ± 0.7	1.8 ± 0.4	2.5 ± 0.5	4.1 ± 1.0
Medium plaque (210 < HU < 550)	24	119 ± 59	1.9 ± 0.4	2.7 ± 0.7	1.7 ± 0.4	2.3 ± 0.5	3.3 ± 0.7
Heavy plaque (HU > 550)	11	245 ± 192	2.1 ± 0.5	2.6 ± 0.5	1.6 ± 0.3	2.1 ± 0.3	2.9 ± 0.6

**Table 5:** ΔLBR obtained when applying PVC in a new image reconstruction (PSF + PVC) and when using the tissue activities obtained with the LP method (LP)

Plaque type	# Lesions	Volume (mm^3^)	ΔLBR_max_ (%)		ΔLBR_mean_ (%)	
			PSF + PVC	LP	PSF + PVC	LP
Non-calcified plaque (HU < 110)	16	499 ± 323	52 ± 34	60 ± 30	43 ± 21	94 ± 32
Light plaque (110 < HU < 210)	15	53 ± 34	59 ± 38	112 ± 51	46 ± 36	137 ± 62
Medium plaque (210 < HU < 550)	24	119 ± 59	41 ± 24	75 ± 28	36 ± 17	101 ± 41
Heavy plaque (HU > 550)	11	245 ± 192	32 ± 30	48 ± 37	33 ± 22	82 ± 42

Table 6 shows the fitting parameters for all the fits in Figure 6 and the Pearson and Spearman correlation coefficients between the measured variables. As expected, the fitting parameter *A* for the LBR_max_ vs HU curves was close to zero when no PVC was applied, demonstrating that the LBR does not depend significantly on the HU value in the PSF images. In contrast, for the PSF + PVC and LP methods, a significant dependence was observed. A similar behavior was found in the LBR_max_ vs volume curves. Positive correlations were found between the LBR/ΔLBR and the segmented volume or HU of the plaque in the PSF images. However, for the PSF + PVC images and the LP method, the correlation was found to be negative and significantly higher for the LP method than for the PSF + PVC images. The higher values for the Spearman correlation factor in the LP curves confirm the non-linear behavior of these curves. Significant differences between the PSF and the PSF + PVC mean LBR were found (*t*-statistic = −12.3, *P* = 5 × 10^−19^) and between the PSF + PVC and the LP mean LBR values (*t*-statistic = −6.1, *P* = 3 × 10^−8^).

**Table 6:** Fitting parameters for the fits performed in Figure 6, and Pearson (*R*) and Spearman (*S*) correlation parameters for the data evaluated

Evaluation	Method	Fitting parameters		Pearson correlation (*R*)	Spearman correlation (*S*)
		*A*	*B*		
LBR vs segmented volume	PSF	−0.023 ± 0.010	2.14 ± 0.09	0.234	0.253
	PSF + PVC	0.018 ± 0.016	2.90 ± 0.15	−0.104	−0.055
	LP	0.048 ± 0.019	2.73 ± 0.16	−0.450	−0.544
LBR vs HU	PSF	0.015 ± 0.030	1.90 ± 0.12	0.356	0.251
	PSF + PVC	0.12 ± 0.05	2.34 ± 0.19	−0.154	−0.156
	LP	0.22 ± 0.06	2.63 ± 0.26	−0.381	−0.443
ΔLBR vs segmented volume	PSF + PVC	0.49 ± 0.19	38.8 ± 7.3	−0.298	−0.264
	LP	4.2 ± 0.8	28.4 ± 7.0	−0.578	−0.680
ΔLBR vs HU	PSF + PVC	5.1 ± 2.2	25.6 ± 8.9	−0.374	−0.337
	LP	10.3 ± 3.2	42 ± 13	−0.495	−0.462

## Discussion

In this work, we assess the ability and usefulness of PVC for PET imaging of plaque-type lesions. Based on phantom and patient data, we are able to demonstrate that the LBR of plaque-type lesions increases by up to 475% and 227% in phantoms and patients, respectively, when adopting a PVC method that is based on a previously proposed methodology.^19^ The demonstrated improvements in LBR should be seen in the light of recent studies by Derlin et al. and Fiz et al., who advocate the use of 18F-NaF PET imaging for the detection and characterization of vulnerable plaques.^7^,^10^,^11^

Of note, the clinical implementation of the LP method differed from the pre-clinical version evaluated in previous work.^19^ Here, the LP method was implemented only as a post-processing step which, together with the additional STIR reconstruction, results in a PVC image. In the pre-clinical implementation, the LP method was also implemented within the reconstruction process (PVC reconstruction).^19^ In this work, we decided to evaluate a version of the LP method that would be easier to implement in clinical practice since it is based on a single post-processing step. This approach should make the algorithm more useful in clinical practice, where many retrospective studies do not have the projection data available and not all institutions have access to their own reconstruction algorithm. As we showed in our pre-clinical implementation, PVC reconstruction approaches can be implemented within the STIR reconstruction framework by calling the LP algorithm after each iteration.

The validation of our PVC method was performed by means of acquisition of a NEMA IQ phantom and a human-sized thorax phantom with three plaque-type lesions. Of note, the PSF matrix of the system must be known in order to ensure the best possible performance of the PVC method. Here, we made the assumption that the PSF can be described by a uniform Gaussian function, which is reasonable when the primary structures of interest (e.g., carotids) are located near the center of the PET transaxial field of view (FOV). The Full Width Half Maximum (FWHM) of the Gaussian blurring was obtained by fitting the LP tissue activities for the 37-mm sphere of the NEMA IQ phantom. As expected, the resulting FWHM values were different for the OSEM (FWHM ~ 8 mm) and PSF (FWHM ~4 mm) reconstructions. The spatially invariant PSF approximation used in this study may not work properly for lesions located far away from the center of the transaxial FOV, given the spatial variance of the PSF.^32^ In consequence, for lesions located far from the center of the FOV, we expect a reduced accuracy of the LP method. This limitation can be solved with a more accurate description of the PSF.

Both NEMA and thorax phantom experiments showed a significant improvement in quantification accuracy of the lesions when the PVC was applied in OSEM or PSF images (Figures 3, 4; Tables 2, 3). The LBR values for each lesion were closer to the reference in the PVC images. For lesions below 10 mm diameter, total recovery of the PVE was not achieved (Tables 2, 3), thus giving LBR values from the PVC images or from the LP tissue activities well below the experimental values. This is mainly due to two reasons: First, the PET-based segmentation will be significantly bigger than the real size of the lesion due to the spread of activity of these very small sources. Second, for these lesions, the Nyquist sampling condition (lesion sizes bigger than 3 voxels in each spatial direction, voxel size 2.03 mm) is not satisfied, and, therefore, a full recovery of the lesion activities using the LP method is not possible.^19^ Nonetheless, large activity recovery values were obtained when applying the PVC method. These values could be further enhanced by reducing the voxel size in the PET image and by using a more accurate segmentation from a high-resolution anatomical image.

In general, the measured LP tissue activity ratios were more accurate than those obtained after the additional reconstruction with STIR. While the lesion contrast always appeared to be quantitatively improved in the PVC image (compared to OSEM or PSF alone), the best quantitative results were nevertheless generally obtained using the directly computed LP activity values with no further image reconstruction process.

The generally higher LBR values observed in the PSF reconstructions are more likely due to Gibbs artifacts (overshoots around the boundaries of hot lesions or organs), which are usually inherent to reconstruction algorithms with resolution recovery.^32^,^33^ We observed that the proposed PVC method corrected for the overestimation of activities, giving LBR closer to the experimental values in the PSF + PVC images and in the LP tissue activities obtained from the PSF images (Table 2).

The performance of the LP method for different segmentation algorithms was evaluated with the NEMA IQ phantom acquisition (Table 2). In general, slightly higher LBR values were observed when the PET threshold-based segmentation was used during PVC. This behavior was more pronounced for smaller spheres. This is due to the fact that the segmented volume of the spheres, using the 50% threshold adapted to background, was always smaller than the actual volume of the sphere (about 70-80% of the actual volume). This is in concordance with similar studies in the literature.^34^ However, for the plaque-type lesions in the thorax phantom and the smallest non-calcified plaques in the patients, as their size is similar to the voxel size, the LP method using a PET-based segmentation is expected to under-perform the LP method using a CT-based segmentation, due to the spread of activity of very small hot sources.

In patient images, no significant correlations were observed between ^18^F-NaF plaque uptake in the uncorrected images and CT-based calcifications. However, in the new reconstructed images with PVC, a significant correlation of ^18^F-NaF uptake and calcification density of the atherosclerotic plaque was observed, due to the better quantitative evaluation of ^18^F-NaF uptake in the calcified plaques (Table 6). Furthermore, the quantification and inverse correlation further improved when considering the tissue activity values obtained with the LP method (Table 6). As expected, the effect of the PVC was more significant in small calcifications, thus resulting in higher ΔLBR for the smaller light plaque lesions (Figure 6). The non-calcified plaques evaluated in this work were relatively large in size (Tables 4, 5). This result in relatively low ΔLBR values, compared with the ones obtained for light plaque lesions.

For the calcified plaques, a CT-based segmentation of the local VOI was used for the PVC. A limitation of this approach, as for the majority of PVC techniques, is that it can be affected by miss-registration errors.^35^ We attempted to limit such errors by carefully verifying the spatial alignment of the PET and CT images prior to applying the PVC. In non-calcified lesions, a PET threshold-based segmentation of the local VOI was used for the PVC. This method works well provided the lesion is clearly visible in the non-corrected image and, therefore, can be segmented accurately. This was the case for the non-calcified lesions analyzed in this work.

### Limitations and Future Work: Evaluation of Coronary Plaque

The main limitation of the method is that non-calcified lesions must be present and detectable on either PET or CT in order for the proposed PVC to be applicable. Another potential limitation of this study is the possibility of non-compensated patient motion during the acquisitions, which could also affect the co-registration of the PET and CT images, and the quantitative properties of the PET image. However, we consider involuntary motion effect to be of minor importance for this evaluation, considering the short PET acquisition time (2 minute per bed position).

Although pathological features in carotid or aortic arteries are likely representative of general atherosclerotic disease, it is known that most of the deaths from cardiovascular events result from complications of inflammatory plaques in the smaller coronary plaques.^36^ The application of the PVC method for the evaluation of 18F-NaF uptake in vulnerable plaque in the coronaries, however, represents a more challenging problem, due to the presence of respiratory and cardiac motion, which have to be compensated before applying the PVC.^27^ The evaluation of an image reconstruction framework, which incorporates motion-compensation (MoCo) and partial volume correction (PVC) for 18F-NaF PET imaging of vulnerable plaque in the coronary arteries, is currently work in progress, with first promising results in simulated data.^37^

## New Knowlegde Gained

We have demonstrated the need for partial volume correction when imaging small structures of interest, such as atherosclerotic plaques in the carotid and aortic arteries by means of PET. PVC can lead to increased LBR_max_ values up to a factor of 3 in small plaque lesions.

## Conclusion

The good performance of the LP-based PVC method was confirmed in both OSEM and PSF reconstructions of phantom data and clinical patients. LBR of plaque-type lesions increased by up to 475% (phantom) and 227% (patients) when PVC was applied. Results from this study following PVC further support reports of an inverse correlation of ^18^F-NaF uptake in calcified plaques with plaque density.

### Electronic supplementary material

Below is the link to the electronic supplementary material.

Supplementary material 1 (PPTX 2851 kb)

## Appendix 1: Description of the LP method

Consider a reconstructed image with a detected lesion (for instance, an atherosclerotic plaque that shows increased uptake of the administered radiotracer). Our goal is to improve the quantification of the activity concentration in that lesion and removing spill-over activity from neighboring tissues. If we have a precise segmentation of organs and tissues, we can take advantage of this information and improve PET quantification based on the following:

- It is reasonable to assume that voxels belonging to the same tissue have a more similar activity than voxels belonging to different tissues. In consequence, voxels from different tissues would be represented by different average activity values.
- We can also estimate the PET counts assigned to the inside of the VOI which actually originated from outside the VOI.

With these assumptions, the measured data, λ_*i*_, can be modeled as

4 $$ \uplambda_{i} = \mathop \sum \limits_{j = 1}^{J} A_{j} P_{ij} + g_{out,i}, $$

where λ_*i*_ are the expected counts per sinogram bin, *i*, *A*_*j*_ the activity for each segmented tissue *j* within the VOI, *P*_*ij*_ is the resolution-blurred tissue shape function for tissue *j* and sinogram bin *i*, and $g_{{{\text{out}},i}}$ represents the background counts coming from the region outside the VOI.

The joint likelihood of measuring a given projection data set, *n*, is given by the product of the Poisson probability density function for each measured projection ray, *i*, over all rays traversing the VOI:

5 $$ L = \mathop \prod \limits_{i \in VOI} \left[ {\frac{{e^{{ - \uplambda_{i} }} \uplambda_{i}^{{n_{i} }} }}{{n_{i} !}}} \right], $$

where $n_{i}$ are the measured counts per LOR. Taking the logarithm of the likelihood, we obtain

6 $$ \ln \left( L \right) = \mathop \sum \limits_{i} n_{i} \ln \left( {\uplambda_{i} } \right) - \uplambda_{i} - \ln \left( {n_{i} !} \right). $$

If we substitute the λ_*i*_ values with Eq. (4), we obtain

7 $$ \ln \left( L \right) = \mathop \sum \limits_{i} n_{i} \ln \left( {\mathop \sum \limits_{j = 1}^{J} A_{j} P_{ij} + g_{{{\text{out}},i}} } \right) - \left( {\mathop \sum \limits_{j = 1}^{J} A_{j} P_{ij} + g_{{{\text{out}},i}} } \right) - \ln \left( {n_{i} !} \right). $$

The tissue activities *A*_*j*_ are determined by maximizing the log-likelihood [Eq. (6)] for the expected value λ_*i*_. This can be done by taking derivatives of *ln(L)* with respect of each tissue activity *A*_*j*_ and setting the results to zero:

8 $$ A_{j}^{\left( k \right)} = \mathop \sum \limits_{j' = 1}^{J} \left[ {\mathop \sum \limits_{i} \frac{{P_{{ij^{'} }} P_{ij} }}{{\uplambda_{i}^{\left( k \right)} }}} \right]^{ - 1}  \mathop \sum \limits_{i} \frac{{P_{ij} \left( {n_{i} - g_{{{\text{out}},i}}^{\left( k \right)} } \right)}}{{\uplambda_{i}^{\left( k \right)} }}. $$

Note that the equations in Eq. 8 cannot be solved analytically for the unknown *A*_*j*′_. Instead, an iterative solution for these equations is sought. Note that the values of $\uplambda_{i}$, $g_{{{\text{out}},i}},$ and *A*_*j*_ are updated in each iteration (*k*).

In this work, we obtain *A*_*j*_ using the following procedure:

1. Segment a VOI (containing the lesions of interest) from the registered anatomical image. Project these segmented volumes, with unity activity voxel values, through an accurate forward model to obtain *P*_*ij*_ matrix elements which represent the contribution of segmented tissue j (with unit activity concentration) to the i’th PET line of response.
2. Mask (zero) the segmented VOI from the reconstructed image, using the anatomical segmentation performed in step 1, and re-project through the same forward model to obtain *g*_out_.
3. Compute all of the necessary matrix elements, $D_{j}$ and $H_{{jj^{\prime}}}$, which are simply short-hand expressions for collections of terms that arise from maximization of the log-likelihood:
  9 $$ H_{{jj^{'} }}^{\left( k \right)} = \mathop \sum \limits_{i} \frac{{P_{{ij^{'} }} P_{ij} }}{{\uplambda_{i}^{\left( k \right)} }} $$
  10 $$ D_{j}^{(k)} = \mathop \sum \limits_{i} \frac{{P_{ij} (n_{i} - g_{out,i}^{(k)} )}}{{\uplambda_{i}^{(k)} }}. $$
4. From the matrix elements calculated previously, estimate the values of tissue activities by inverting the matrix H to solve equation 1, obtaining
  11 $$ A_{j}^{(k)} = \mathop \sum \limits_{{j^{'} = 1}}^{J} \left[ {H_{jj'}^{(k)} } \right]^{ - 1} D_{j}^{(k)}. $$
5. The procedure above is repeated from step 3 using new estimates of the $A^{(k)}_{j}$ to compute new elements of matrices H and D, and, after that, improved estimates of the $A^{(k)}_{j}$. This procedure is repeated until the estimates change by less than a very small amount in a single iteration. The convergence of the method is usually very fast, achieving convergence within two to three iterations.

The obtained tissue activities (*A*_*j*_) can be used as a prior in a new reconstruction procedure to obtain a locally “Partial Volume Corrected (PVC)” PET image, as detailed in Figure 1. Note that the LP method conserves the total number of counts in the image, as it just re-assigns counts from one segmented region to another, seeking for consistency between the tissue segmentation and the PET activity distribution.

## Abbreviations

PET: Positron emission tomography
CT: Computed tomography
CVD: Cardiovascular disease
PVE: Partial volume effect
PVC: Partial volume correction
LP: Local projection
LBR: Lesion-to-background ratio
ΔLBR: Relative change of lesion-to-background ratio
OSEM: Ordered subsets expectation maximization
PSF: Point spread function
