# Supplementary material for: Partial volume correction for improved PET quantification in 18F-NaF imaging of atherosclerotic plaques
Source: J Nucl Cardiol. 2017 Feb 7;25(5):1742–56. doi: 10.1007/s12350-017-0778-2 (PMC6153866; doi:10.1007/s12350-017-0778-2)
Supplement: Supplementary file 1 — Supplementary material 1 (PPTX 2851 kb) [file 12350_2017_778_MOESM1_ESM.pptx]

## Slide 1
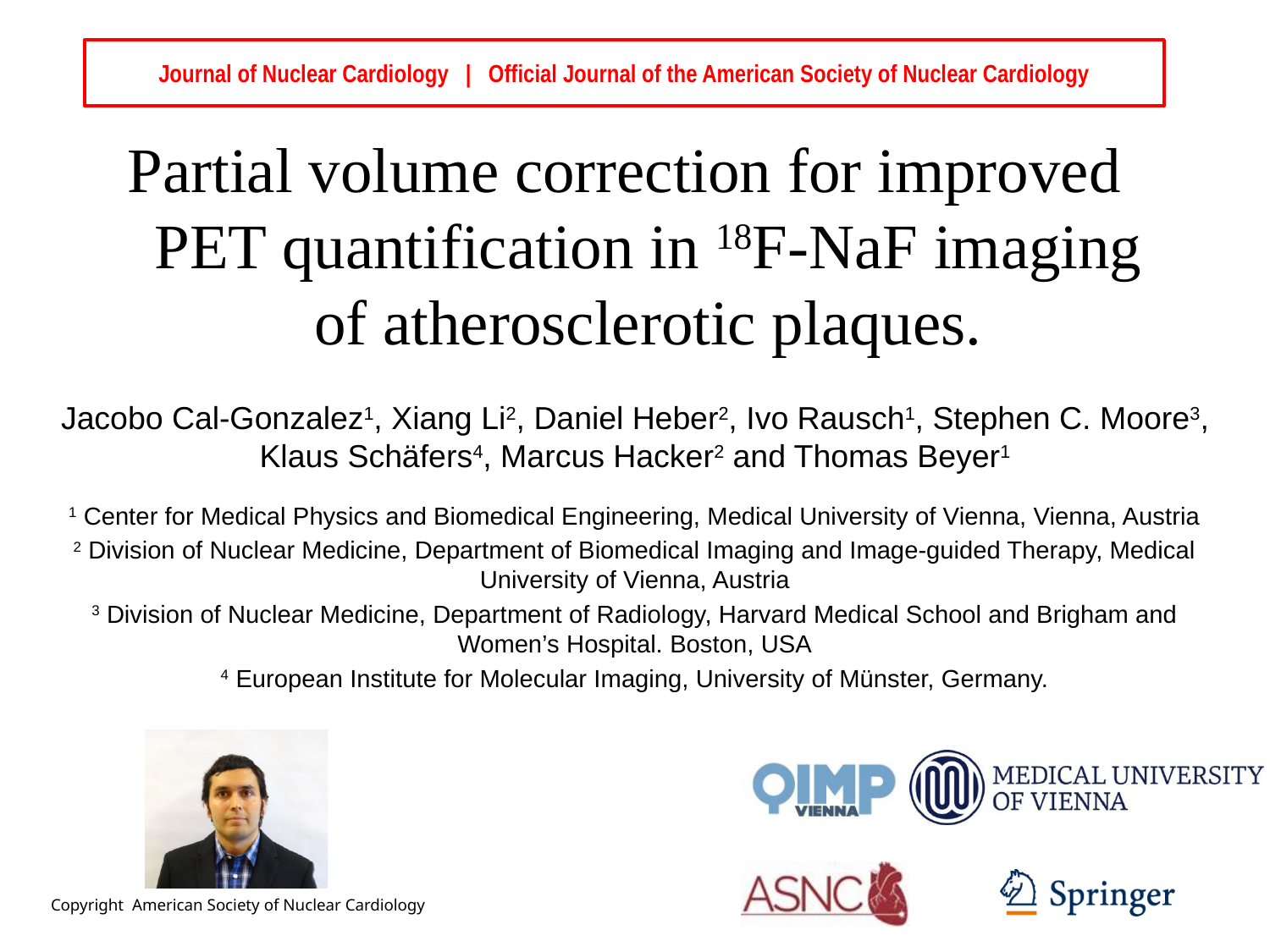

Journal of Nuclear Cardiology | Official Journal of the American Society of Nuclear Cardiology
# Partial volume correction for improved PET quantification in 18F-NaF imaging of atherosclerotic plaques.
Jacobo Cal-Gonzalez1, Xiang Li2, Daniel Heber2, Ivo Rausch1, Stephen C. Moore3, Klaus Schäfers4, Marcus Hacker2 and Thomas Beyer1
1 Center for Medical Physics and Biomedical Engineering, Medical University of Vienna, Vienna, Austria
2 Division of Nuclear Medicine, Department of Biomedical Imaging and Image-guided Therapy, Medical University of Vienna, Austria
3 Division of Nuclear Medicine, Department of Radiology, Harvard Medical School and Brigham and Women’s Hospital. Boston, USA
4 European Institute for Molecular Imaging, University of Münster, Germany.
Copyright American Society of Nuclear Cardiology

## Slide 2
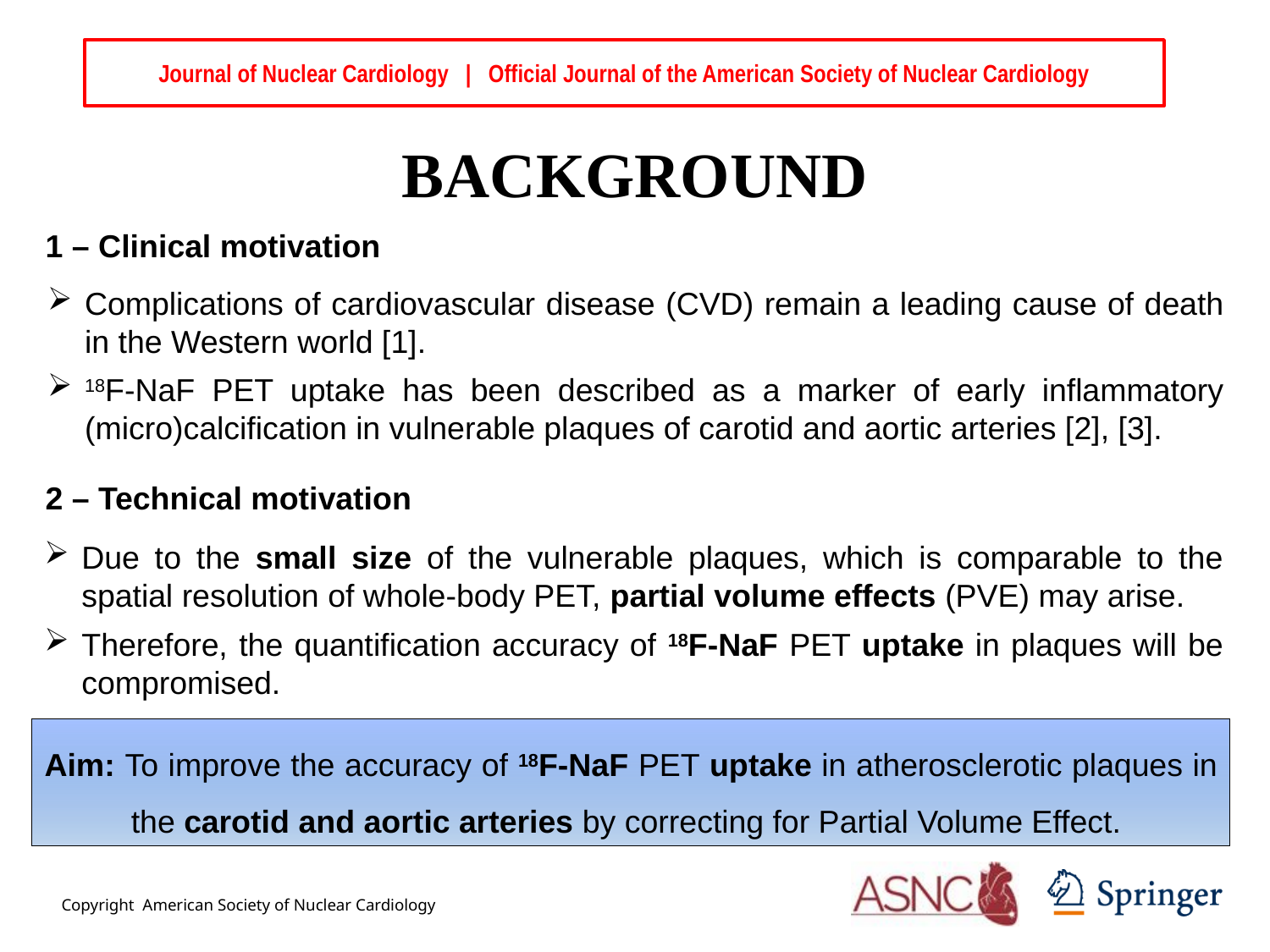

Journal of Nuclear Cardiology | Official Journal of the American Society of Nuclear Cardiology
# BACKGROUND
1 – Clinical motivation
Complications of cardiovascular disease (CVD) remain a leading cause of death in the Western world [1].
18F-NaF PET uptake has been described as a marker of early inflammatory (micro)calcification in vulnerable plaques of carotid and aortic arteries [2], [3].
2 – Technical motivation
Due to the small size of the vulnerable plaques, which is comparable to the spatial resolution of whole-body PET, partial volume effects (PVE) may arise.
Therefore, the quantification accuracy of 18F-NaF PET uptake in plaques will be compromised.
Aim: To improve the accuracy of 18F-NaF PET uptake in atherosclerotic plaques in the carotid and aortic arteries by correcting for Partial Volume Effect.
Copyright American Society of Nuclear Cardiology

## Slide 3
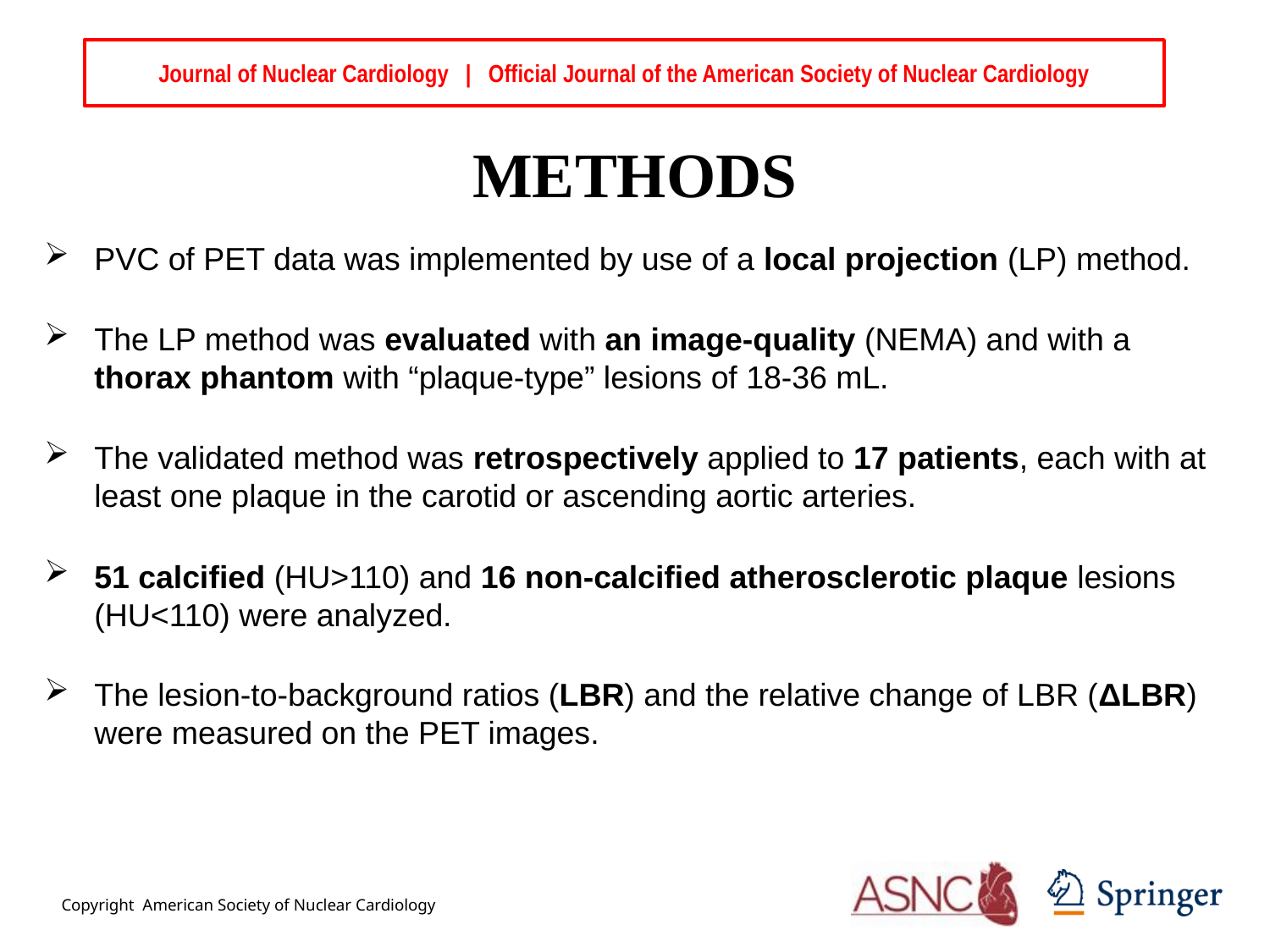

Journal of Nuclear Cardiology | Official Journal of the American Society of Nuclear Cardiology
# METHODS
PVC of PET data was implemented by use of a local projection (LP) method.
The LP method was evaluated with an image-quality (NEMA) and with a thorax phantom with “plaque-type” lesions of 18-36 mL.
The validated method was retrospectively applied to 17 patients, each with at least one plaque in the carotid or ascending aortic arteries.
51 calcified (HU>110) and 16 non-calcified atherosclerotic plaque lesions (HU<110) were analyzed.
The lesion-to-background ratios (LBR) and the relative change of LBR (ΔLBR) were measured on the PET images.
Copyright American Society of Nuclear Cardiology

## Slide 4
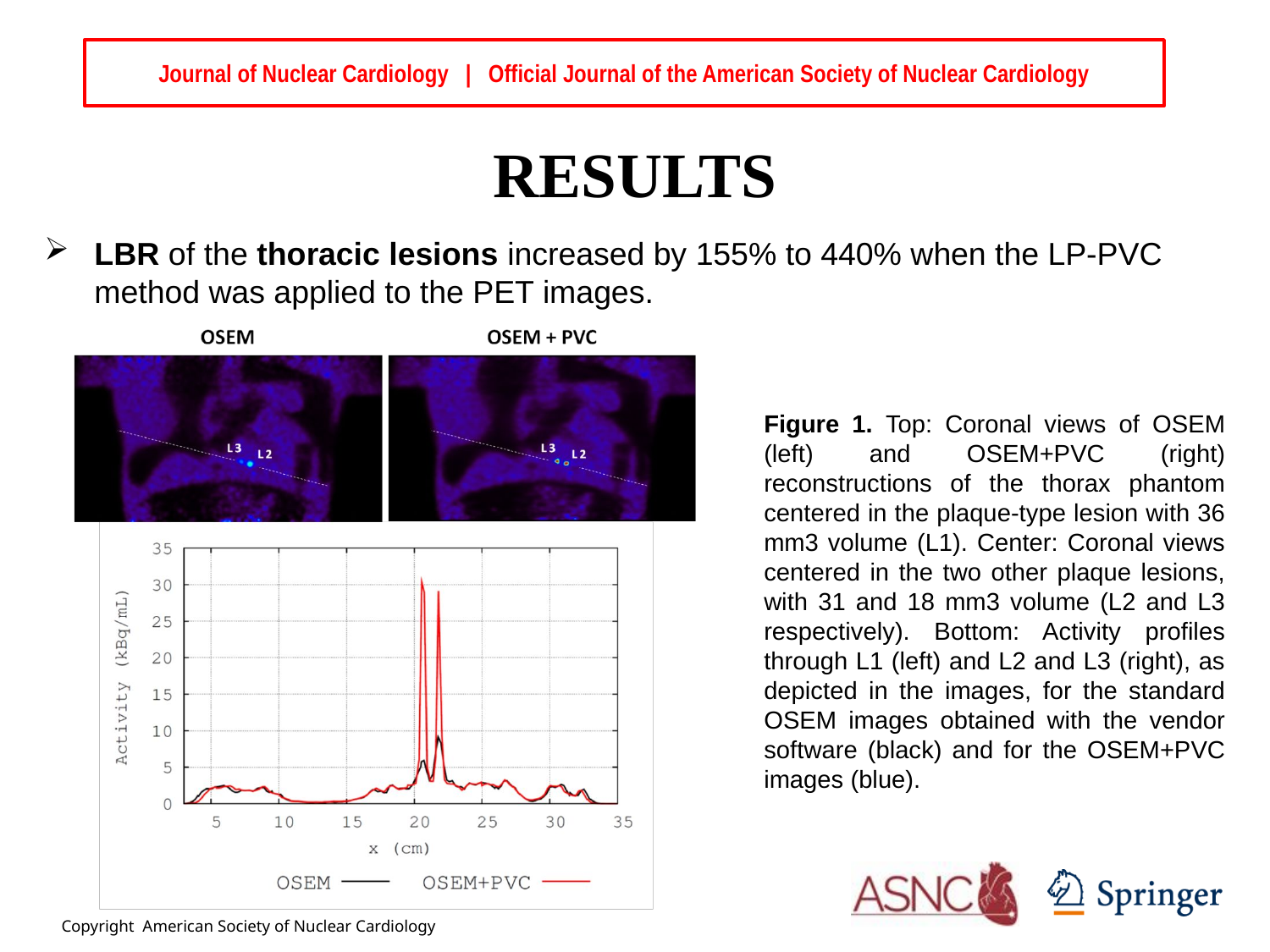

Journal of Nuclear Cardiology | Official Journal of the American Society of Nuclear Cardiology
# RESULTS
LBR of the thoracic lesions increased by 155% to 440% when the LP-PVC method was applied to the PET images.
Figure 1. Top: Coronal views of OSEM (left) and OSEM+PVC (right) reconstructions of the thorax phantom centered in the plaque-type lesion with 36 mm3 volume (L1). Center: Coronal views centered in the two other plaque lesions, with 31 and 18 mm3 volume (L2 and L3 respectively). Bottom: Activity profiles through L1 (left) and L2 and L3 (right), as depicted in the images, for the standard OSEM images obtained with the vendor software (black) and for the OSEM+PVC images (blue).
Copyright American Society of Nuclear Cardiology

## Slide 5
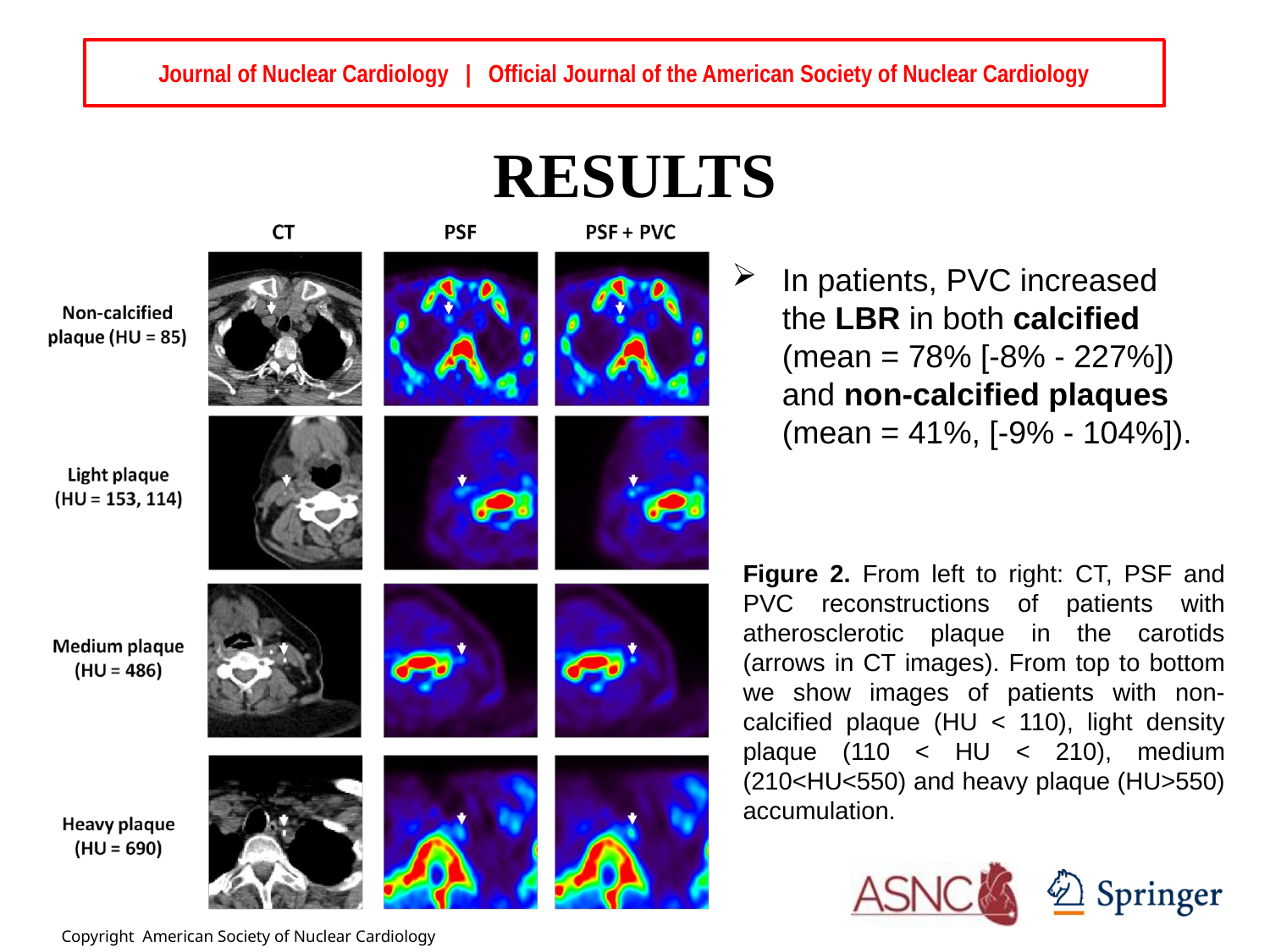

Journal of Nuclear Cardiology | Official Journal of the American Society of Nuclear Cardiology
# RESULTS
In patients, PVC increased the LBR in both calcified (mean = 78% [-8% - 227%]) and non-calcified plaques (mean = 41%, [-9% - 104%]).
Figure 2. From left to right: CT, PSF and PVC reconstructions of patients with atherosclerotic plaque in the carotids (arrows in CT images). From top to bottom we show images of patients with non-calcified plaque (HU < 110), light density plaque (110 < HU < 210), medium (210<HU<550) and heavy plaque (HU>550) accumulation.
Copyright American Society of Nuclear Cardiology

## Slide 6
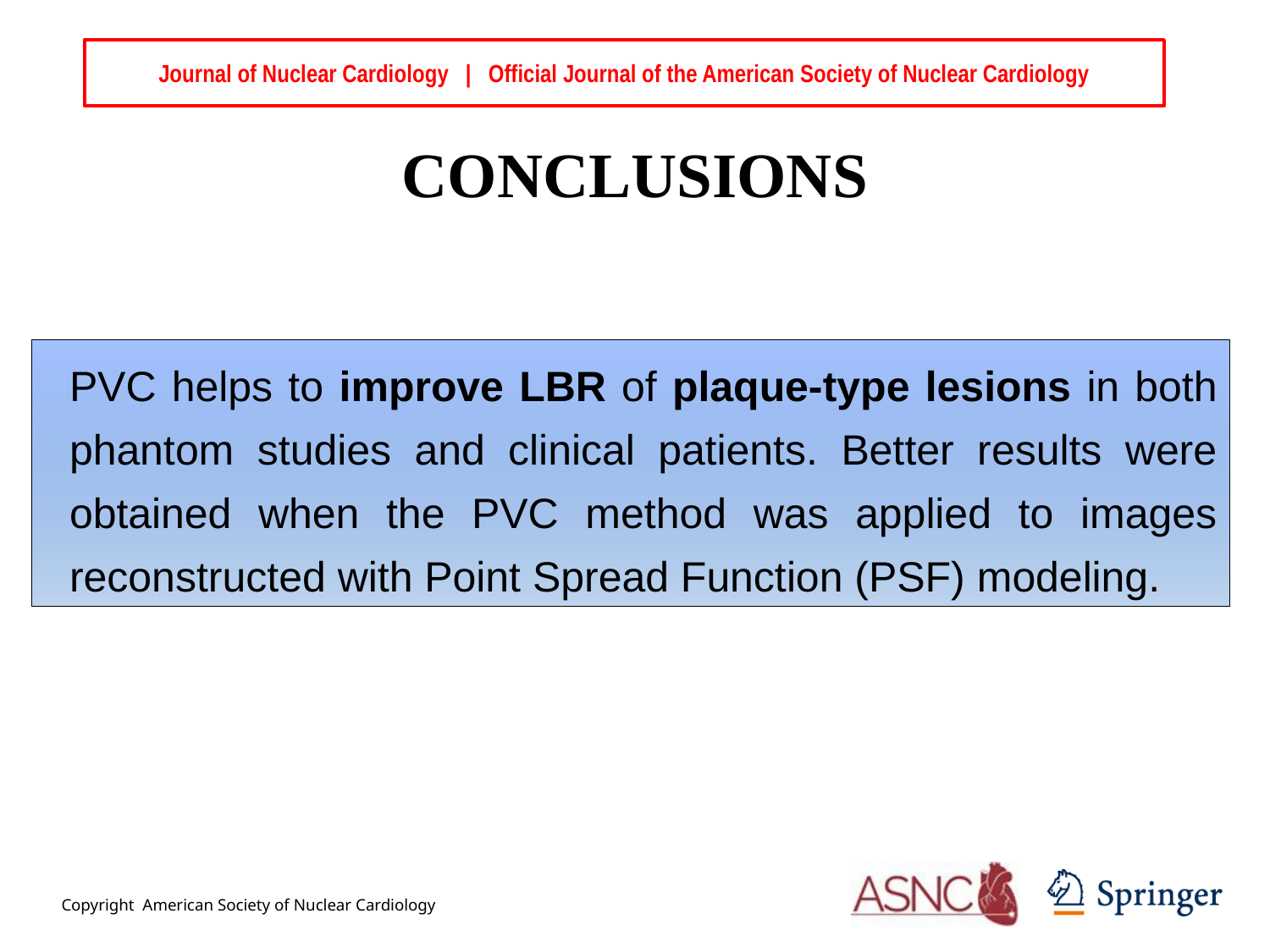

Journal of Nuclear Cardiology | Official Journal of the American Society of Nuclear Cardiology
# CONCLUSIONS
PVC helps to improve LBR of plaque-type lesions in both phantom studies and clinical patients. Better results were obtained when the PVC method was applied to images reconstructed with Point Spread Function (PSF) modeling.
Copyright American Society of Nuclear Cardiology
